# PB.39. CT or not CT: a systematic review of the breast on CT of the chest and abdomen over 1 year - another screening tool?

**DOI:** 10.1186/bcr3725

**Published:** 2014-11-03

**Authors:** S Flais, A Newland, A Flais, B Ellis

**Affiliations:** 1 Ealing Hospital, Southall, UK

## Introduction

Images of breast tissue are usually included on CT scans of the chest or abdomen. It is generally thought that CT has a low sensitivity for breast pathology. We tested this assumption to assess the importance of the breast as a review area on CT.

## Methods

All CT scans which included some breast tissue and were performed on female patients over a 1-year period at our institution were retrospectively reviewed by a breast radiologist. The breast tissue was scrutinised and, if required, an addendum added to the report and sent urgently to the referring clinician. If the patient was referred for breast assessment, standard imaging findings were compared with the CT appearances.

## Results

A total of 2,500 CT scans were reviewed. Thirty per cent of the scans provided imaging of the whole breast. Addendums were added to 112 reports; a recall rate of 5.4%. Sixty-three patients were never referred for breast imaging. The average time from addendum to assessment was 3 months. Forty-nine patients were assessed with breast imaging. Twenty-nine patients underwent a biopsy. Eleven patients had a malignancy. One had a papilloma upgraded to DCIS at surgery.

## Conclusion

Significant abnormalities in the breast are visible on CT, and are frequently overlooked. These results have been fed back to our general radiology colleagues and there has been a noticeable increase in reported abnormalities within the breast. We aim to reassess the impact of this work with further audit, which will hopefully demonstrate a reduced rate of missed findings in this area.

